# Development of Nanocomposite Film Comprising of Polyvinyl Alcohol (PVA) Incorporated with Bacterial Cellulose Nanocrystals and Magnetite Nanoparticles

**DOI:** 10.3390/polym13111778

**Published:** 2021-05-28

**Authors:** Naphat Usawattanakul, Selorm Torgbo, Prakit Sukyai, Somwang Khantayanuwong, Buapan Puangsin, Preeyanuch Srichola

**Affiliations:** 1 Biotechnology of Biopolymers and Bioactive Compounds Special Research Unit, Department of Biotechnology, Faculty of Agro-Industry, Kasetsart University, Chatuchak, Bangkok 10900, Thailand; naphat.pop@gmail.com (N.U.); selorm.t@ku.th (S.T.); 2 Cellulose for Future Materials and Technologies Special Research Unit, Department of Biotechnology, Faculty of Agro-Industry, Kasetsart University, Chatuchak, Bangkok 10900, Thailand; fforsok@ku.ac.th (S.K.); fforbpp@ku.ac.th (B.P.); preeyanuch.anu@ku.th (P.S.); 3 Department of Forest Products, Faculty of Forestry, Kasetsart University, Chatuchak, Bangkok 10900, Thailand; 4 Kasetsart Agricultural and Agro-Industrial Product Improvement Institute, Kasetsart University, Chatuchak, Bangkok 10900, Thailand

**Keywords:** bacterial cellulose nanocrystal, magnetite nanoparticles, nanocomposite film, polymeric nanomaterial, polyvinyl alcohol

## Abstract

Nanocomposite film of poly(vinyl alcohol) (PVA) incorporated with bacterial cellulose nanocrystals (BCNCs) and magnetite nanoparticles (Fe_3_O_4_) is reported in this study. The BCNC-Fe_3_O_4_ nanoparticles and PVA film was prepared by in situ synthesis technique using chemical co-precipitation. Different concentrations of BCNC-Fe_3_O_4_ (20%, 40% and 60% *w*/*w*) were mechanically dispersed in PVA solution to form the nanocomposite film. Transmission electron microscopy (TEM) analysis of BCNC-Fe_3_O_4_ nanoparticles showed irregular particle sizes ranging from 4.93 to 30.44 nm with an average size distribution of 22.94 nm. The presence of characteristic functional groups of PVA, BCNC and Fe_3_O_4_ were confirmed by Fourier transform infrared (FTIR) spectroscopy and X-ray diffraction (XRD) analysis. Scanning electron microscope (SEM) attached energy dispersive spectroscopy (EDS) and vibrating sample magnetometer (VSM) analysis revealed that, the iron content and magnetic property increased with increasing BCNC-Fe_3_O_4_ content. The saturation magnetizations (*MS*) value increased from 5.14 to 11.56 emu/g. The PVA/ BCNC-Fe_3_O_4_ at 60% showed the highest Young’s modulus value of 2.35 ± 0.16 GPa. The prepared film could be a promising polymeric nanomaterial for various magnetic-based applications and for the design of smart electronic devices.

## 1. Introduction

Magnetite nanoparticles (Fe_3_O_4_) are stimuli responsive materials that have gained much attention from researchers worldwide. This is because of its properties such as nanosize, crystallinity, large specific surface area, superparamagnetism and magnetization value [1]. Fe_3_O_4_ have been studied for many technological applications including magnetic resonance imaging, ferrofluids for audio speakers, magnetic recording media, magnetic targeted drug delivery, magnetic hyperthermia, separation and removal of contaminants [1,2] and nucleic acid separation [3]. However, the pristine magnetic nanoparticles are chemically active, and can easily oxidize in air to other forms leading to loss in magnetic properties and dispersibility. Additionally, due to the nano nature of the particles, it may lead to environmental pollution if used without functionalizing to restrict the particles from being blown by air [4]. Furthermore, in acidic conditions, the iron oxide may lose its magnetic property due to its susceptibility to acid. Thus, coating layers may be required to keep the magnetic properties for application in diverse fields [5,6]. In this regard, Yantasee et al. prepared superparamagnetic iron oxide (Fe_3_O_4_) nanoparticles functionalized with thiol as an effective sorbent material for toxic substances, which have affinity to iron oxide lattices [7]. The thiol-modified magnetic nanomaterial was also reported for gold recovery from dilute solutions [8]. However, the use of synthetic compound as a conjugant for magnetic materials in matrices may pose a lot of environmental challenges.

Recent studies have focused on the design of various nano-adsorbents containing magnetic particles for removal of metal ions for various applications such as wastewater treatment, extraction of genomic DNA, magnetic resonance imaging, drug delivery systems, separation, preconcentration of various anions and cations and bioseparation [9,10]. These may be due to the unique characteristics of the nanoparticles pertaining to those applications, which includes the selective and high adsorptive abilities of metal ions and biomolecules, easy and fast production, rapid uptake and easy separation of the magnetic adsorbent through an external magnetic field [11]. The use of magnetic green adsorbents based on natural polymers are gaining ground as good biomaterial with strong adsorption properties [12]. They are also environmentally friendly, sustainable and naturally renewable [13]. Previous studies have been successfully achieved by introducing various polymers such as biodegradable chitosan nanofibers [14], gelatin [15], amino-functionalized [5], dextran [16,17], pullulan [18] and starch [19]. Kloster et al. reported of a magnetic composite film based on alginate as convenient and efficient natural polymer-based adsorbents [12]. Aminodextran-coated magnetic nanoparticles and graphene oxide have also been reported for cellular magnetic resonance imaging [20]. Although many natural polymers have been studied as magnetic-composite materials, none of these studies have considered cellulose nanocrystals (CNCs) from naturally pure and non-toxic bacterial cellulose (BC).

In the present study, BCNCs were conjugated with magnetite nanoparticles in PVA to produce magnetic hybrid nanocomposite film. BCNC is a rigid shape and highly crystalline particle, which was obtained in our previous studies through acid hydrolysis of bacterial cellulose [21]. It has the same properties as cellulose nanocrystals (CNCs) [21,22], and has been utilized in the development of highly biocompatible and biodegradable materials for various applications. The BCNC is non-toxic, biocompatible with rich hydroxyl group and has high surface area appropriate for combining it with other materials [22]. Unlike plant, which requires pretreatment and bleaching before cellulose extraction and subsequent hydrolysis to obtain CNCs, the pristine BC is naturally pure and does not require further treatment and bleaching after purification from culture medium. The PVA, on the other hand, is a synthetic polymer used for various applications in different industries. It is soluble in water, effective in film forming, has emulsifying and adhesive quality, is biocompatible, has good swelling, is non-toxic and is non-carcinogenic [23,24]. Reddy et al. synthesized nanocomposite of PVA integrated with nickel sulphide nanoparticles for potential application in energy storage devices [25]. PVA filled with zinc sulphide nanoparticles nanocomposite films [26] and PVA with cadmium sulphide nanocomposites [27] have also been reported with enhanced dielectric properties. Furthermore, PVA, polyvinylidene fluoride (PVDF) and nafion composites have been prepared by solution casting for application in sensors, fuel cells and batteries [28]. For the purposes of adsorption and separation, PVA has been used in protein adsorption [29,30], immobilization of lipase [31], nanocomposite device for bioseparation [32], selective adsorption and separation of dyes [33] and gas separation [34]. However, using pristine Fe_3_O_4_ in PVA film could lead to agglomeration, a property that has been reported to affect the “Van der Waals forces and the pull of magnetic dipole” [35]. Polymer coating may be required to promote uniform distribution of Fe_3_O_4_ particles. Therefore, adding BCNC will stabilize and enhance distribution of Fe_3_O_4_ in PVA to open up its applications.

Despite the prominent features of BCNC and its hydroxyl group as binding site for ions, to the best of knowledge, no studies have been done on using it alongside PVA and Fe_3_O_4_ for consideration in the separation process and smart electronic devices. This study used BCNC to enhance distribution of Fe_3_O_4_ in PVA film with improved mechanical and superparamagnetic properties. The synthesized nanofilm is magneto-responsive and can be manipulated via an external magnetic field. The high hydrophilicity of BCNC coupled with the superparamagnetic property of the Fe_3_O_4_ nanoparticle, makes it applicable in diverse fields for bioseparation and adsorption of various molecules and pollutants. This could also serve as an environmentally friendly, sustainable and naturally renewable green adsorbents and material for smart electronic devices.

## 2. Materials and Methods

### 2.1. Materials 

*Komagataeibacter xylinus* (TISTR 975) strains was purchased from the Thailand Institute of Scientific and Technological Research, Bangkok, Thailand. Ferrous sulfate heptahydrate (FeSO_4_·7H_2_O, Ajax Finechem Pty., Ltd., Auckland, New Zealand), ferric chloride hexahydrate (FeCl_3_·6H_2_O, PanReac AppliChem Barcelona, Spain), and poly(vinyl alcohol) (PVA, Mw = 89,000–98,000) (EMD Millipore Corporation, Darmstadt, Germany) were use in the study. All other reagents used were of analytical grade.

### 2.2. Preparation BC and Extraction of BCNC

The BCNC used in this study was previously extracted and reported in one of our studies [21]. For BC preparation, 5% (*w*/*v*) of sucrose and 2.5% (*w*/*v*) of ammonium sulfate were added to coconut water (100 mL) and sterilized by boiling at 100 °C. After cooling to room temperature, 5% glacial acetic acid was added as an acidifier to pH 4.5. *Komagataeibacter xylinus* (10% *v*/*v*) was then inoculated and left under static condition at room temperature for 5 days to form bacterial cellulose of 5 mm thickness. The cellulose pellicles were washed and boiled in 1% (*w*/*v*) NaOH solution for 15 min at 100 °C and finally boiled in deionized (DI) water to attain a neutral pH (pH = 7). After purification, excess water was removed by compressing the BC and then grinded into powder. The powdered BC was hydrolyzed by treating with 60% (*w*/*w*) sulfuric acid using the BC to acid ratio of 1:20 g/mL with continuous stirring at 45 °C. The hydrolysis reaction was stopped by adding distilled water. The sample was subsequently centrifuged at 13,000 rpm for 15 min at 4 °C and then washed three times to remove acid residuals. Dialysis was employed to neutralized the BCNC sample and kept in refrigerator for the next stage of the experiment [21]. The BCNC sample was sonicated for 30 min before being used in further studies.

### 2.3. Preparation of BCNC and Fe_3_O_4_ Powder

The Fe_3_O_4_ and BCNC composite was prepared by in situ coprecipitation under ultrasonic irradiation following the method by [36] with modifications. In summary, 0.125 mol (3.375 g) of ferrous sulfate heptahydrate (FeSO_4_·7H_2_O) and 0.25 mol (6.9575 g) of ferric chloride hexahydrate (FeCl_3_·6H_2_O) with Fe^2+^ and Fe^3+^ ions in the 1:2 M ratio were dissolved in 50 mL (25 mL each) distilled water. BCNC suspension of 50 mL (0.0353 g dry matter/mL) was mixed with the resultant iron solution and shook for 12 h under an incubator shaker with 250 rpm at room temperature. After that, 100 mL of aqueous NH_4_OH was added under the ultrasonic irradiation at 60 °C for 30 min in an ultrasonic bath (Elmasonic S 100 H, Singen, Germany), operating at 50/60 Hz with a power of 550 W under vacuum using the circulating aspirator (Sibata, Model: WJ-20, Tokyo, Japan). Finally, the prepared sample was rinsed with absolute ethanol and DI water, followed by drying in the oven at 60 °C for at least 6 h before being grinded into powder. The prepared BCNC and Fe_3_O_4_ composite was thereafter labeled “BM”.

### 2.4. Preparation of PVA Film Incorporated with BM

The PVA (Mw = 89,000–98,000) concentration of 10 g/100 mL was dissolved on a hot plate using a magnetic stirrer, and kept in an oven at 90 °C for 12 h to have a homogeneous solution. The nanocomposite film was prepared using digital overhead mixer (IKW rw 20) by heating the PVA solution on a hot plate at 90 °C. Different concentrations of BM (20%, 40% and 60% *w*/*w*) were added and mixed at 200 rpm for 30 min. The resultant hydrogel mixture was gently poured into a petri dish with an average diameter of 14 cm. The spirit level was used to fix the petri dish to ensure uniform surface and even distribution of the hydrogel, then allowed to settle for 30 min at room temperature to form a film. The film was finally dried in an oven at 40 °C for 12 h. 

### 2.5. Characterizations 

Transmission electron microscope (TEM) operated at an accelerating voltage of 80 kV (JEM-2100Plus, Tokyo, Japan) was used to observe the morphology and determine particles size of BM.

The SEM-EDS (Oxford instrument, X-Max, Tehran, Iran) operated at an accelerating voltage of 10 kV was used to observe the atomic composition of iron and elemental mapping of the film. 

The chemical interaction of the materials in the film was recorded using the Fourier transform infrared spectrometer (Bruker model Tensor 27, New York, NY, USA) at room temperature using the attenuated total reflectance (ATR) mode. 

The crystallinity and characteristic fingerprint of the composite films were measured using an X-ray diffractometer (Bruker D8 Advance, Rosenheim, Germany) with Cu- Kα radiation and operated at a voltage of 40 kV. The scattered radiation was detected at the scan rate of 2°/min from 2θ = 5–70°. 

The magnetic responsive behavior of pure Fe3O4 and their corresponding nanocomposite films, were evaluated using a vibrating sample magnetometer (Model M2000/2100) following [37].

The mechanical properties of composite films were determined according to ASTM D882-02 standard test method using a universal testing machine (Shimadzu model AGS5kN, Kyoto, Japan) as described by [38] with modifications. The films with average dimension of 10 mm × 50 mm were fitted with a 5 kN load cell, with a crosshead speed of 10 mm/min and 3 mm distance between clamps. The analysis was done in triplicate, and the results were presented as an average value of each film. 

### 2.6. Statistical Analysis

The data collected were subjected to the analysis of variance (ANOVA) using GenStat software 12th edition. The multiplicative comparison of means was statistically performed with a Bonferroni test. The experiments were performed with *n* = 3 replicates and data presented as the average of three replicates.

## 3. Results and Discussion

### 3.1. Morphological and Particle Size Distribution 

**Figure 1:**
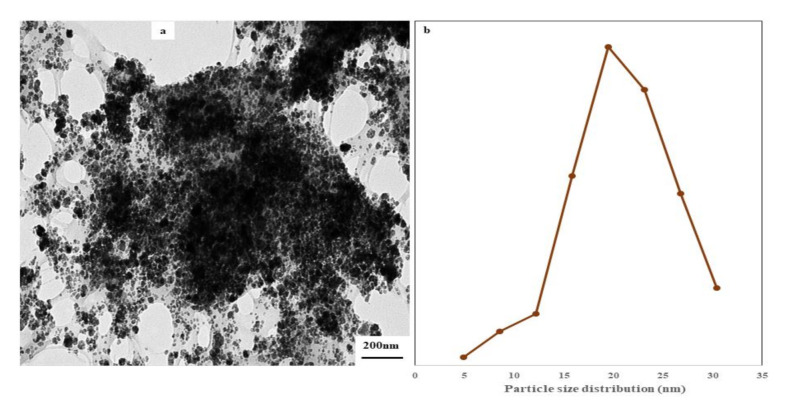
TEM image of BCNC mixed with Fe_3_O_4_ (BM) powder (**a**) and particle size distribution (**b**) of nanoparticles.

Figure 1 shows the TEM image and particle size distribution of BM as nanopowder before using in the PVA matrix. Figure 1a shows an irregular particles sizes ranging from 4.93 to 30.44 nm with an average size distribution of 22.94 nm (Figure 1b). Prior to the synthesis of BM, the shape and size of BCNC and Fe_3_O_4_ nanoparticles were analyzed by atomic force microscopy, which presented varied morphologies of BCNC and Fe_3_O_4_ (Supplementary Materials Figure S1). The BCNC nanoparticles showed rod-like shapes with an average size of 23.57 nm, whiles the Fe_3_O_4_ showed spherical shapes with average dimension of 4.42 nm. From the TEM result, it could be deduced that the binding of the Fe3O4 nanoparticles on BCNC resulted in an increase in particle size. The processing of the two nanomaterials after synthesis through mechanical grinding might have caused the irregularities in shape. The magnetic nanoparticles occur predominantly in cubic and spherical forms. The cubic nanoparticles had lower surface anisotropy due to their flat surface and disordered spins, while the spherical nanoparticles had higher surface anisotropy as a result of their curved surface with more spin surface canting [39]. The surface disordering of spins, either as a result of processing method or cladding by BCNC, could cause reduction in saturation magnetization of magnetic particles. 

### 3.2. SEM-EDS Analysis

**Figure 2:**
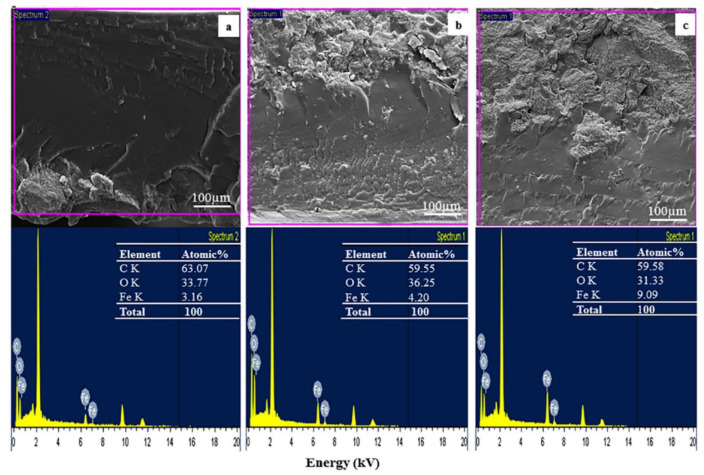
EDS analysis of (**a**) PVA/BM20, (**b**) PVA/BM40 and (**c**) PVA/BM60 composite films.

The analysis of surface morphology of the films by SEM shows non porous surface with no significant difference between the PVA and the composites films (Figure S2). The presence of Fe_3_O_4_, specifically iron concentration in the composite film, was analyzed by EDS. The sample was prepared by breaking the film in liquid nitrogen and the spectrometry was taken at the break point. The results presented in Figure 2 show varied concentrations of iron in the various composite films, which confirm the presence of Fe_3_O_4_ in the films, while the carbon and oxygen atoms depicted the PVA and BCNC, respectively. As expected, the film with the highest BM content (PVA/BM60) showed the highest percentage atomic peak of 9.09% for iron in the film (Figure 2c), followed by PVA/BM40 (4.20%), while PVA/BM20 showed the lowest atomic peak value of 3.16% (Figure 2a). This confirms successful dispersion and bonding of BCNC and Fe_3_O_4_ in the PVA film. This was possible through the formation of a covalent bond between the magnetic particles and hydroxyl group or carboxylic group on the surface of BCNC and PVA. This will serve as a chelating ligand to improve the adsorbing ability of the magnetic composite film with multiple binding sites [40].

**Figure 3:**
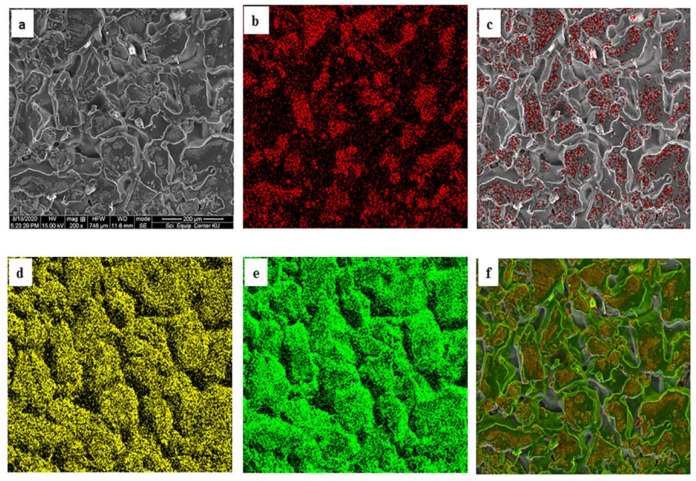
SEM micrograph (**a**) PVA/BM60 (200×), EDS elemental mapping of (**b**) Fe (red), (**c**) PVA/BM60_Fe mix, (**d**) O (yellow), (**e**) C (green) and (**f**) PVA/BM60_Fe_O_C mix composite film.

**Figure 4:**
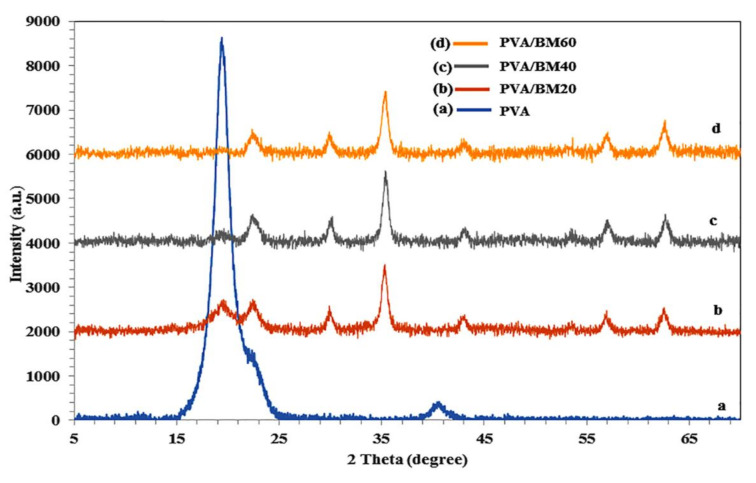
X-ray diffraction pattern of (**a**) PVA, (**b**) PVA/BM20, (**c**) PVA/BM40 and (**d**) PVA/BM60 composite films.

**Figure 5:**
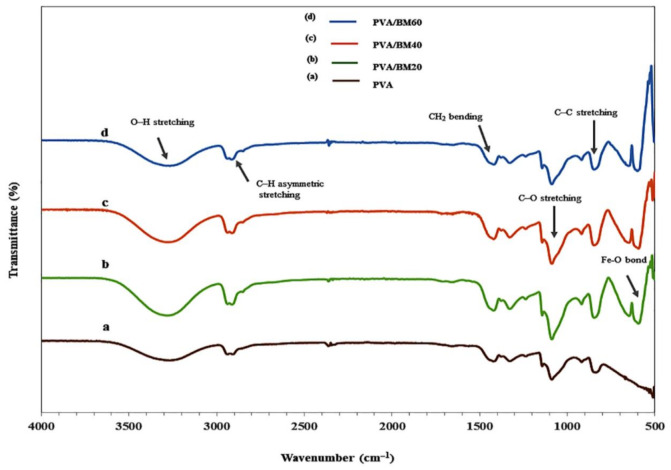
FTIR spectroscopy of (**a**) PVA, (**b**) PVA/BM20, (**c**) PVA/BM40 and (**d**) PVA/BM60.

The presence and distribution of BM nanoparticles in the PVA matrices was authenticated in the PVA/BM60 film using SEM-EDS elemental smart mapping. The results in Figure 3a shows the distribution of the nanoparticles within the PVA matrices. The various elements present in the nanoparticles (BM) were depicted by different colors as red (iron), yellow (oxygen) and green (carbon) (Figure 3b–e). Figure 4c shows the distribution of Fe in the PVA matrix. It is of interest to note that the Fe was not added to the PVA in isolation but in combination with BCNC, therefore, its distribution in the polymer indicated the stabilizing effects of BCNC. This was established in Figure 3f, where the three elements were mapped in the composite film. The uniform distribution of O and C in the film was laudable because the two elements were the main component of PVA and BCNC and O from Fe_3_O_4_. The nanoparticles may be distributed in the polymers through chemical bonding between the various atoms (O, C and Fe) in the nanoparticles and the functional groups of PVA [41]. The O–H group of PVA formed hydrogen bond with the OH and C-H group of BCNC, which contained Fe_3_O_4_. This interaction within the composite film was further established by FTIR analysis (Figure 5).

### 3.3. XRD Analysis

The diffraction pattern of the composite films with the characteristic fingerprints of the polymeric materials and magnetic nanoparticles are presented in Figure 4. The fingerprints reveal the chemical interaction of hydrogen bonds between nanoparticles and the PVA matrix [42]. The BCNC possessed a higher amount of hydroxyl groups and the PVA matrix, thus resulting in strong filler–matrix bonding in the film. The crystalline diffraction peaks of PVA are present at 2θ = 19.4°, 22.1° and 40.4° (Figure 4a). The peak at 2θ = 19.4° corresponded to an orthorhombic lattice of PVA, which specifies its crystalline nature. However, the incorporation of the nanoparticles resulted in a drastic decrease of the PVA peak intensity in the composite films. The presence of Fe_3_O_4_ in the film was confirmed by its fingerprint peaks at 2θ = 30°, 35.6°, 43.2°, 57.2° and 62.9°, which corresponded with the crystal plane of (220), (311), (400), (511) and (440), respectively, based on the reference standard peak (JCPDS no. 019-0629) [43,44]. The crystalline peak of BCNC is observed at 2θ = 22.6°, which corresponded to the (002) crystallographic plane, while the other minor peaks disappeared due to chemisorption of Fe ions by the hydroxyl group to form Fe-OH molecules. The intensity of PVA peaks continuously declined with increasing BM content in the PVA chains. The crystalline nature of the film was due to the strong intermolecular interaction between PVA chains through intermolecular hydrogen bonding. Nonetheless, the wide interplanar spacing could be attributed to the weak self-hydrogen bonding between PVA chains [45], which enabled successful interaction with BM in the composite films.

### 3.4. FTIR Analysis

To further interpret the intermolecular interactions of PVA with BCNC and Fe_3_O_4_**,** FTIR analysis was conducted to identify the various functional groups as presented in Figure 5. The pure PVA shows a wide absorption band traversing 3518–3122 cm^−1^, which is ascribed to the bonded hydroxyl (O–H stretching) vibration in the crystalline phase due to the extensive H-bond [46]. The absorption peaks at 2914, 1415, 1080 and 839 cm^−1^ were apportioned to C–H asymmetric stretching, CH_2_ bending, C–O stretching and C–C stretching, respectively. The incorporation of BM resulted in a slight broadening and increased intensity of the absorption peaks associated with O–H stretching at 3556–3072, 3533–3122 and 3535–3130 cm^−1^ for PVA/BM20, PVA/BM40 and PVA/BM60, respectively. This may be attributed to the intermolecular bonding between the hydroxyl groups in PVA and BCNC [46]. However, the band at 2914 cm^−1^ for C–H asymmetric stretching remained the same with slight increase in peak intensity in the composite films. The slight changes recorded in the composite films could be attributed to the chemical interactions between the functional groups of the PVA matrix and BCNC, which served as a binder between PVA and Fe_3_O_4_. The successful integration of BCNC and Fe_3_O_4_ nanoparticle in PVA was confirmed by the presence of two new absorption bands at 653.86 and 586.36–597.92 cm^−1^ in only the composite films, which were ascribed to Fe-O-C bonds between iron oxides and polymers. The fingerprint of Fe_3_O_4_ in the range of 586.36–597.92 cm^−1^ indicates the presence of Fe-O bond of Fe_3_O_4_ [47]. The appearance of characteristic fingerprints of the various materials demonstrated successful synthesis of the ternary composite film.

### 3.5. Vibrating Sample Magnetometer (VSM) 

**Figure 6:**
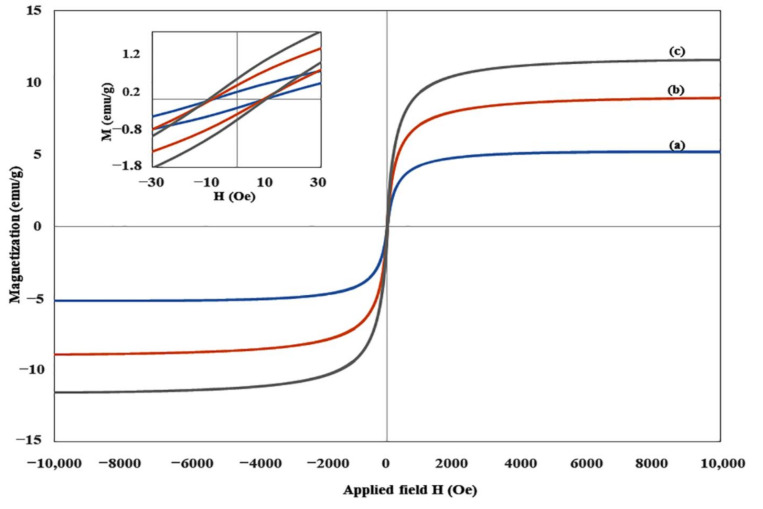
Field-dependent magnetization (***M*** versus ***H***) curves of (**a**) PVA/BM20, (**b**) PVA/BM40 and (**c**) PVA/BM60.

The magnetic behavior of the films, which is a critical parameter of magnetic composites was evaluated by VSM. The VSM analysis was carried out and the results of hysteresis curves are presented in Figure 6. The saturation magnetization (*MS*) value was derived from the VSM measurement at room temperature with the magnetic field of ±10 kOe. The result shows a direct proportional increase in *MS* with increasing BM in the film. The PVA/BM60 composite film recorded the highest *MS* of 11.56 emu/g, followed by PVA/BM40 (8.91 emu/g), while PVA/BM20 recorded 5.14 emu/g. The *MS* values obtained in PVA/BM40 and 60 were higher than values reported in related studies [48,49,50]. The insert in Figure 6 shows the hysteresis loop upon the reversal of the applied magnetic field. The PVA/BM60 sample showed the highest amount of energy dissipation (hysteresis loop) compared with the other samples. This implies the ability of the film to retain a large fraction of the saturated magnetic field when the driving field was removed. The saturation magnetization is a function of temperature in the bulk magnetic material at low temperatures, therefore the high value of *MS* in PVA/BM60 is undeniably because of the high amount of BM present in the film. The films showed superparamagnetic properties with minimal remanent magnetization (*Mr*) values of 0.212, 0.378 and 0.536 emu/g for PVA/BM20, PVA/BM40 and PVA/BM60, respectively. It is clear from the study that increasing the BM content will definitely increase the magnetic behavior of the film for the intended purpose. This is in agreement with previous studies where increasing Fe_3_O_4_ concentration (magnetic content) in the composite material, increases the sensitivity of the material to changes in external magnetic field with a subsequent increase in magnetic values [12,51,52]. However, an overdose of Fe_3_O_4_ content may lead to agglomeration, which may impede its dispersion in the matrix and uniform distribution of its magnetic moment. The superparamagnetic property and *MS* behavior of the film implies it could be responsive to alternating current magnetic fields for magnetic separation and adsorption of molecules and design of smart magnetic devices.

### 3.6. Mechanical Strength 

**Figure 7:**
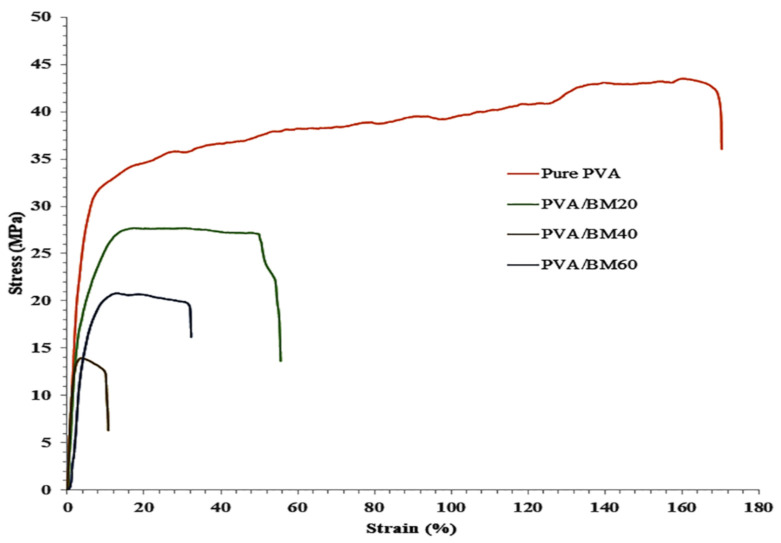
Stress–strain curve showing the break point of pure PVA, PVA/BM20, PVA/BM40 and PVA/BM60.

The tensile test was performed to calculate the Young’s modulus in order to determine the effect of the BCNC and Fe_3_O_4_ on the PVA film in terms of the maximum stress that a material can withstand and stiffness of the composites. The results presented in Table 1 show high significant difference (*p* ≤ 0.05) between pure PVA and PVA/BM composite films. The pure PVA recorded the highest tensile strength of 41.98 ± 1.80 MPa followed by PVA/BM20. Among the composites, there was a significant difference between PVA/BM20 and PVA/BM40 with tensile values of 24.36 ± 1.70 and 12.72 ± 0.70 MPa, respectively. There was no significant difference between PVA/BM20 and PVA/BM60, which had a tensile value of 19.54 ± 1.53 MPa. In terms of Young’s modulus, there was no significant difference between the pure PVA, PVA/BM20 and PVA/BM40 films, which recorded 1.51 ± 0.15, 1.32 ± 0.12 and 1.58 ± 0.02 GPa, respectively. However, increasing BM concentration to 60% resulted in a significant increase in the Young’s modulus of PVA/BM60 composite film with a value of 2.35 ± 0.16 GPa. The stress–strain curve (Figure 7) showed the percentage elongation at break of the samples. The introduction of BM led to a decrease in the elasticity of the films. The pure PVA had the highest percentage elongation of 169.9% followed by PVA/BM20 (50.8%) and PVA/BM60 (32.0%), while PVA/BM40 recorded the lowest elongation of 10.1%. The decrease in value can be explained by the destruction of the samples at the initial stage of neck propagation as a result of thermomechanical instability of the samples when BM was added [53]. The high tensile strength and elongation at break of the pure PVA is laudable as it is an elastic material that can stretch when pulled before breaking. The decrease in the elasticity of the composite films could be a result of the solid particles inclusion and interactions between the PVA matrix and BM nanoparticles through the formation of mechanical and hydrogen bonds. The reinforced BM nanoparticles caused stiffening of the PVA matrix, which restricted the mobility of PVA molecular chains and subsequent reduction in flexibility of the nanocomposite films [42,54]. This was evident in the Young’s modulus results, where the PVA/BM60 showed the highest stiffness. This is better than values reported in other literature [50,55].

## 4. Conclusions

The design of this study saw successful development of ternary magnetic nanoparticle and polymeric nanocomposite (PVA/BM) film. TEM analysis of BM showed irregular particles with an average size distribution of 22.94 nm. The XRD and FTIR analysis showed the presence of functional groups of PVA, BCNC and Fe_3_O_4_ in the composite films. The distribution of the nanoparticles in the PVA film was confirmed by smart mapping of the elements present in the nanoparticles. Increasing the BM content resulted in an increase in the magnetic properties and Young’s modulus of the film. The *MS* exhibited by PVA/BM60 with superparamagnetic property would guarantee an efficient heating under an oscillating magnetic field, which is promising for the design of smart electronic devices. It can also be manipulated by external magnetic fields and reused multiple times in adsorption of molecules. This study serves as the foundation for fabricating an environmentally friendly, sustainable and naturally renewable green adsorbent and smart electronic devices.

## Figures and Tables

**Table 1:** The mechanical properties of pure PVA and PVA/BM composite films.

Sample	Tensile Strength (MPa)	Young’s Modulus (GPa)
Pure PVA	41.98 ± 1.80 ^c^	1.51 ± 0.15 ^a^
PVA/BM20	24.36 ± 1.70 ^b^	1.32 ± 0.12 ^a^
PVA/BM40	12.72 ± 0.70 ^a^	1.58 ± 0.02 ^a^
PVA/BM60	19.54 ± 1.53 ^a,b^	2.35 ± 0.16 ^b^

Values with the same letter means the difference is not statistically significant, while different letters are statistically significant (*p* ≤ 0.05).

## Data Availability

The data presented in this study are available on request from the corresponding author.

## Supplementary Materials

The following are available online at https://www.mdpi.com/article/10.3390/polym13111778/s1, Figure S1. AFM micrograph of (a) bacterial cellulose nanocrystals (1 × 1 µm) and (b) magnetite nanoparticles (2 × 2 µm); Figure S2. SEM micrograph of (a) PVA, (b) PVA/BM20, (c) PVA/BM40 and (d) PVA/BM60 composite films.
