# Supplementary material for: Development of Nanocomposite Film Comprising of Polyvinyl Alcohol (PVA) Incorporated with Bacterial Cellulose Nanocrystals and Magnetite Nanoparticles
Source: Polymers (Basel). 2021 May 28;13(11):1778. doi: 10.3390/polym13111778 (PMC8198786; doi:10.3390/polym13111778)
Supplement: Supplementary file 1 [file polymers-13-01778-s001.zip › polymers-1219445-supplementary.pdf]

polymers

MDPI

Supplementary Materials

Development of Nanocomposite Film Comprising of Polyvinyl Alcohol (PVA) Incorporated with Bacterial Cellulose Nanocrystals and Magnetite Nanoparticles

Naphat Usawattanakul [1], Selorm Torgbo [1,2], Prakit Sukyai [1,2,*], Somwang Khantayanuwong [2,3], Buapan Puangsin [2,3] and Preeyanuch Srichola [2,4]

1 Biotechnology of Biopolymers and Bioactive Compounds Special Research Unit, Department of Biotechnology, Faculty of Agro-Industry, Kasetsart University, Chatuchak, Bangkok 10900, Thailand; naphat.pop@gmail.com (N.U.); selorm.t@ku.th (S.T.)

2 Cellulose for Future Materials and Technologies Special Research Unit, Department of Biotechnology, Faculty of Agro-Industry, Kasetsart University, Chatuchak, Bangkok 10900, Thailand; fforsok@ku.ac.th (S.K.); fforbpp@ku.ac.th (B.P.); preeyanuch.anu@ku.th (P.S.)

3 Department of Forest Products, Faculty of Forestry, Kasetsart University, Chatuchak, Bangkok 10900, Thailand

4 Kasetsart Agricultural and Agro-Industrial Product Improvement Institute, Kasetsart University, Chatuchak, Bangkok 10900, Thailand

* Correspondence: fagipks@ku.ac.th

Citation: Usawattanakul, N.; Torgbo, S.; Sukyai, P.; Khantayanuwong, S.; Puangsin, B.; Srichola, P. Development of Nanocomposite Film Comprising of Polyvinyl Alcohol (PVA) Incorporated with Bacterial Cellulose Nanocrystals and Magnetite Nanoparticles. *Polymers* **2021**, *13*, 1778. https://doi.org/10.3390/polym13111778

Academic Editor: José Ignacio Velasco

Received: 27 April 2021
Accepted: 25 May 2021
Published: 28 May 2021

Publisher's Note: MDPI stays neutral with regard to jurisdictional claims in published maps and institutional affiliations.

Copyright: © 2021 by the authors. Licensee MDPI, Basel, Switzerland. This article is an open access article distributed under the terms and conditions of the Creative Commons Attribution (CC BY) license (http://creativecommons.org/licenses/by/4.0/).

1. AFM Analysis

AFM was used to characterize the morphology of the dimensional image of BCNC and Fe_3O_4 samples. The measurements were performed in tapping mode using an Asylum model MFP-3D AFM (Bio, USA) at ambient temperature. The dimension of the sample was measured by Gwyddion software.

2. Morphological and Dimensional Characterization

The microscopy analysis of the shape and size of BCNC and Fe_3O_4 nanoparticles are represented in Figure S1. The micrographs presented varied morphologies of BCNC and Fe_3O_4. The BCNC nanoparticles showed a rod-like shape with average size of 23.57 nm, whiles the Fe_3O_4 showed a spherical shape with average dimension of 4.42 nm.

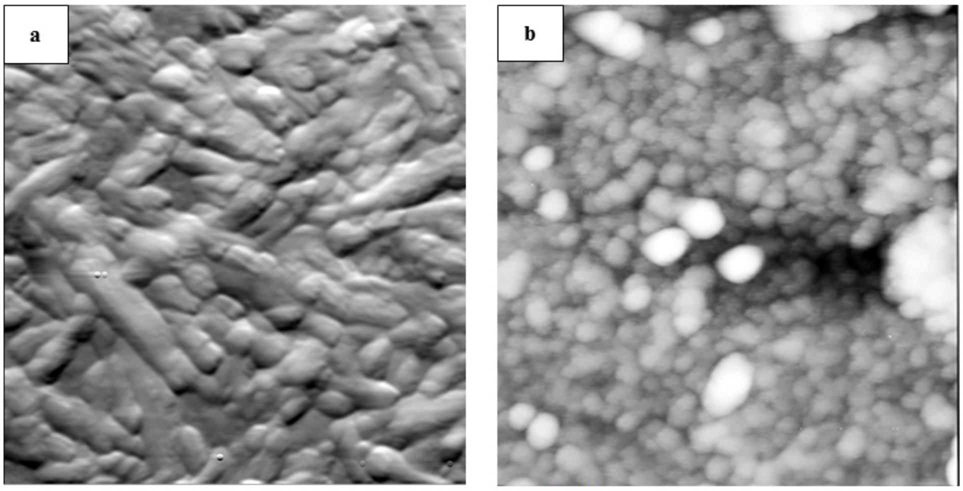

Figure S1. AFM micrograph of (**a**) bacterial cellulose nanocrystals (1 × 1μm) and (**b**) magnetite nanoparticles (2 × 2 μm).

3. SEM Image

The SEM analysis of surface morphology of the films showed non porous surface with no significant difference between the neat PVA and the composites films (Figure S2). The composite films surfaces are dense with small nodular structures which may be due to homogeneous dispersion of nanoparticles in the PVA film and strong intermolecular hydrogen bonding between PVA matrices and nanoparticles.

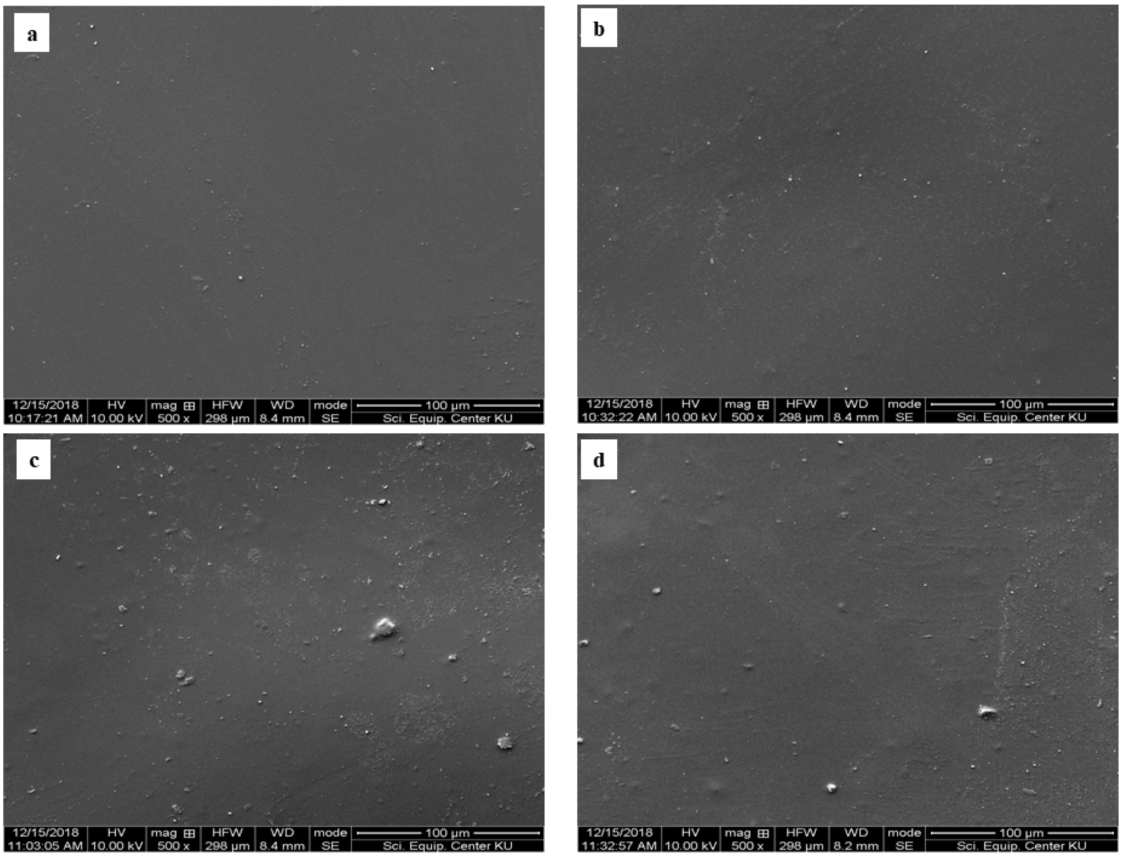

Figure S2. SEM micrograph of (**a**) PVA, (**b**) PVA/BM20, (**c**) PVA/BM40 and (**d**) PVA/BM60 composite films.
